# Application of AI-based virtual standardized patients in physician-patient communication training: a study based on the SEGUE framework

**DOI:** 10.3389/fpubh.2026.1768518

**Published:** 2026-03-31

**Authors:** Ning Sun, Xintong Zhou, Zhongqian Yang, Yu Zhou, Ruihang Ma, Zhong Wang

**Affiliations:** 1 National Key Laboratory of Frigid Zone Cardiovascular Disease and Department of Cardiology, General Hospital of Northern Theater Command, Shenyang, Liaoning, China; 2 Centre for Intelligence-Based Medicine & Policy Advance Clinical Transformation (IMPACT), The Chinese University of Hong Kong, Shenzhen, Guangdong, China; 3 Graduate Student Affairs Office, The Fourth Affiliated Hospital of China Medical University, Shenyang, Liaoning, China; 4 Emergency Medicine Department of Northern Theater Command General Hospital, Shenyang, Liaoning, China; 5 Nursing School, China Medical University, Shenyang, Liaoning, China; 6 Department of Critical Care Medicine, The First Affiliated Hospital of China Medical University, Shenyang, Liaoning, China

**Keywords:** doctor-patient communication, medical education, randomized controlled trial, SEGUE framework, virtual standardized patient

## Abstract

**Background:**

Developing effective doctor–patient communication skills is a critical component of medical education. The SEGUE framework offers a structured and systematic approach for teaching and assessing communication competence. However, traditional standardized patient (SP) training is resource-intensive, time-consuming, and difficult to scale for large student cohorts. AI-based virtual standardized patients (AI-VSPs) have emerged as a promising alternative, providing repeatable, accessible, and scalable training opportunities. This study aimed to evaluate the effectiveness and feasibility of AI-VSPs within SEGUE-based communication training for medical students, compared with traditional SPs.

**Methods:**

In a parallel mixed-methods randomized controlled trial, 82 senior clinical medical students were randomized to train with AI-VSPs (*n* = 40) or traditional SPs (*n* = 42) for 2 h following a 3-week medical ethics course. Communication performance was assessed using SEGUE total scores, Content and Process subscales, training frequency was recorded, and participant feedback was analyzed qualitatively.

**Results:**

Both groups improved significantly in SEGUE scores after training (*P* < 0.05). The AI-VSP group achieved higher total scores (18.53 ± 2.51 vs. 17.33 ± 2.13) and Content scores (12.55 ± 1.91 vs. 11.74 ± 1.38) than the traditional SP group, while Process scores were similar. AI-VSP students completed more training sessions (4.10 vs. 3.79, *P* = 0.03). Qualitative feedback highlighted AI-VSPs' repeatability, convenience, and realistic interactions, with fewer negative comments than traditional SPs.

**Conclusion:**

SEGUE-based simulation training effectively enhances medical students' communication skills. AI-VSPs offer scalable, repeatable, and practical advantages, helping learners complete communication tasks thoroughly and providing targeted feedback, but they do not necessarily improve real-world communication competence such as empathy or relational skills.

## 1 Introduction

Physician–patient communication is one of the core competencies in clinical practice, permeating the entire diagnostic and therapeutic process. The level of a physician's communication skills directly affects clinical outcomes, patient satisfaction, and the incidence of medical disputes (1, 2). The World Federation for Medical Education (WFME), as well as medical education accreditation standards in various countries, identify communication ability as one of the essential competencies for clinical practitioners (3).

However, although the current medical education system in China has explicitly recognized the need for physician–patient communication competency (4), it only offers a limited number of theoretical courses in medical ethics. Specialized practical training in clinical communication has not yet been incorporated into the standard curriculum (5). As a result, medical students entering clinical work or internships possess only rudimentary ethical knowledge and generally lack adequate communication skills—ultimately affecting both their professional performance and patient satisfaction (4).

The SEGUE framework, proposed by Gregory Makoul in 2001 as a systematic communication model for clinical encounters (6), is a structured and operable teaching and assessment tool that offers a systematic approach to communication training in medical education (7–9). As a mature and well-established framework, it has been widely adopted by medical schools in the United States and Canada to support structured communication training for medical students and clinicians (10).

After 2006, the SEGUE framework was introduced into China, but its application has remained largely limited to small-scale research projects and has not been fully incorporated into routine medical education (11–13). One key reason is that the traditional use of SEGUE relies heavily on standardized patients (SPs), which makes large-scale implementation challenging in China. Moreover, SP-based training is cost-intensive, time-consuming, and places substantial burdens on individuals portraying patients, further limiting its feasibility (14, 15). For students, such training is also constrained by time and physical settings, restricting its scalability, particularly in resource-limited regions and institutions.

In recent years, with the development of virtual reality technology and artificial intelligence, Virtual Standardized Patients (VSPs) have emerged as a promising educational tool (16). VSPs can simulate realistic clinical scenarios, present a variety of cases, and enhance immersion through natural language interaction. They are not restricted by time or physical space, can be simultaneously accessed by multiple users, and can be reused repeatedly—making them more cost-effective and practical than human SPs (17).

The present study aims to integrate Virtual Standardized Patient (VSP) technology with the SEGUE framework to develop a scientific and systematic communication training tool. This tool is intended to provide medical students with accessible and reusable training scenarios. In addition, by comparing training outcomes with those of traditional human standardized patients, this study seeks to evaluate the effectiveness of VSP-based training and provide empirical evidence for the digitalization and intelligent development of physician–patient communication education (18).

## 2 Methods

The aim of this study was to compare the training effectiveness of human standardized patients (SPs) and AI-based virtual standardized patients (VSPs) under the SEGUE evaluation framework, and to assess the feasibility and operability of VSPs in communication training. The study adopted a randomized controlled trial (RCT) design and employed a parallel mixed-methods approach, integrating both quantitative and qualitative data collection.

### 2.1 SEGUE framework as the evaluation tool

The SEGUE framework was selected as the primary tool for evaluating communication performance. It is a structured checklist designed to assess physician–patient communication competence and consists of five domains encompassing 25 specific behavioral items.

According to the original SEGUE structure, the 25 items are distributed as follows: Q1–Q5 (Set the Stage), Q6–Q15 (Elicit Information), Q16–Q19 (Give Information), Q20–Q23 (Understand the Patient's Perspective), and Q24–Q25 (End the Encounter). The present study strictly adhered to this original categorization without introducing any additional reclassification.

A Chinese-adapted version of the SEGUE scale, introduced by China Medical University in 2006 (19), was used in this study.

### 2.2 Design of standardized patients

Four representative physician–patient communication scenarios commonly encountered in clinical practice were selected as the basis for constructing standardized patients (SPs): delivering bad news, preoperative consultation, patient anxiety and questioning the treatment plan, and chronic disease management. To enhance generalizability, existing medical records, audio–visual communication data, and clinical expertise were integrated to develop SPs corresponding to internal medicine, surgery, obstetrics and gynecology, and pediatrics (Table 1).

**Table 1:** Design of standardized patients for medical communication training.

VSP ID	Department	Gender	Age	Diagnose	Communication scenario	Training focus	Basis for corpus design
1	Internal medicine	Male	58	Advanced lung cancer	Delivering bad news	Communicate unfavorable diagnosis appropriately and provide emotional support to the patient	Real lung cancer medical records and doctor–patient communication videos
2	Surgery	Male	45	Cholelithiasis (planned laparoscopic cholecystectomy)	Preoperative consultation	Explain the purpose, risks, and expected outcomes of the surgery; confirm informed consent	Surgical preoperative communication templates and clinical physicians' experience
3	Gynecology	Female	32	Polycystic ovary syndrome (PCOS)	Patient anxiety and doubts about treatment plan	Explain the principles, course, and side effects of the treatment; alleviate patient anxiety and distrust	Real outpatient communication recordings and clinical physicians' experience
4	Pediatrics	Female (mother of patient)	35	Childhood asthma	Chronic disease management	Guide the parent on long-term management, medication adherence, and follow-up plans	Pediatric chronic disease follow-up case videos

To ensure compatibility with the SEGUE assessment framework, communication behavior scoring criteria were developed according to the objectives of each SEGUE dimension. These criteria were designed to ensure that each SP could adequately cover and trigger all dimensions and specific items during the interaction (Table 2).

**Table 2:** SEGUE-based design framework for the AI virtual standardized patient.

Dimension	Objective task	Communication behavior standards/scoring points
Set the Stage (S)	Establish a communication context and create a trusting atmosphere as the foundation for physician–patient interaction.	Focuses on whether the physician uses polite greetings, clearly introduces the purpose and process of communication, builds a trusting relationship, and protects the patient's privacy.
Elicit Information (E)	Systematically understand the patient's condition, needs, and background through effective questioning and active listening.	Focuses on whether the physician uses open-ended questions to explore the patient's condition, pays attention to emotional responses, demonstrates empathy and care, and displays medical literacy and professional knowledge.
Give Information (G)	Clearly and accurately convey medical information to the patient and verify their understanding.	Focuses on whether the physician communicates information in clear and concise language, ensures patient comprehension, provides reassurance with empathy and patience, and encourages the patient to ask questions.
Understand the Patient's Perspective (U)	Recognize and respond to the patient's emotions, values, and expectations to build psychological resonance.	Focuses on whether the physician uses empathetic language to express understanding of the patient's feelings, actively listens and addresses concerns, and demonstrates empathy and humanistic care.
End the Encounter (E)	Summarize the communication content, establish a clear follow-up plan, and conclude the interaction positively.	Focuses on whether the physician summarizes key points, clarifies the next steps in treatment, and ends the encounter with warmth, respect, gratitude, and encouragement.

#### 2.2.1 Traditional (human) standardized patients

Medical records, communication transcripts, and video materials from real patient encounters between March and June 2025 were collected as the source material. For each SP scenario, 10 cases were selected based on information richness and suitability for role-play. With reference to expert opinions from corresponding clinical specialties, common communication patterns were extracted, and standardized scripts were created according to each SEGUE item (Appendices 1–4).

Four students with relevant acting experience (matching gender with the SP profiles) were recruited and trained based on the scripts, including patient background, dialogue content, emotional and physical reactions, and alert points. Training continued until all actors were able to perform their roles consistently and reproducibly, without prompting or leading statements.

#### 2.2.2 AI-based virtual standardized patients (AI-VSPs)

The same patient source materials, background information, and standardized scripts used for the human SPs were employed to construct the AI-based virtual standardized patients (AI-VSPs). The AI-VSPs were implemented using the DeepSeek-chat large language model (R1 version at the time of the study), accessed via a cloud-based application programming interface (API). The model was used in its base form and was not fine-tuned with study-specific data.

To ensure stability, reproducibility, and alignment with educational objectives, AI-VSP behavior was controlled through structured prompt engineering combined with rule-based constraints derived from the SEGUE framework. Specifically, patient demographic characteristics, clinical background, emotional tone, and permissible information disclosure were predefined. The AI-VSP was instructed to disclose information only when appropriately elicited by the learner, thereby simulating realistic patient response patterns and avoiding proactive prompting.

Manual iterative adjustments were conducted to ensure that each AI-VSP could naturally trigger all SEGUE assessment items through regular conversation, without explicitly guiding students toward specific questions or behaviors.

#### 2.2.3 Consistency assessment

**Figure 1:**
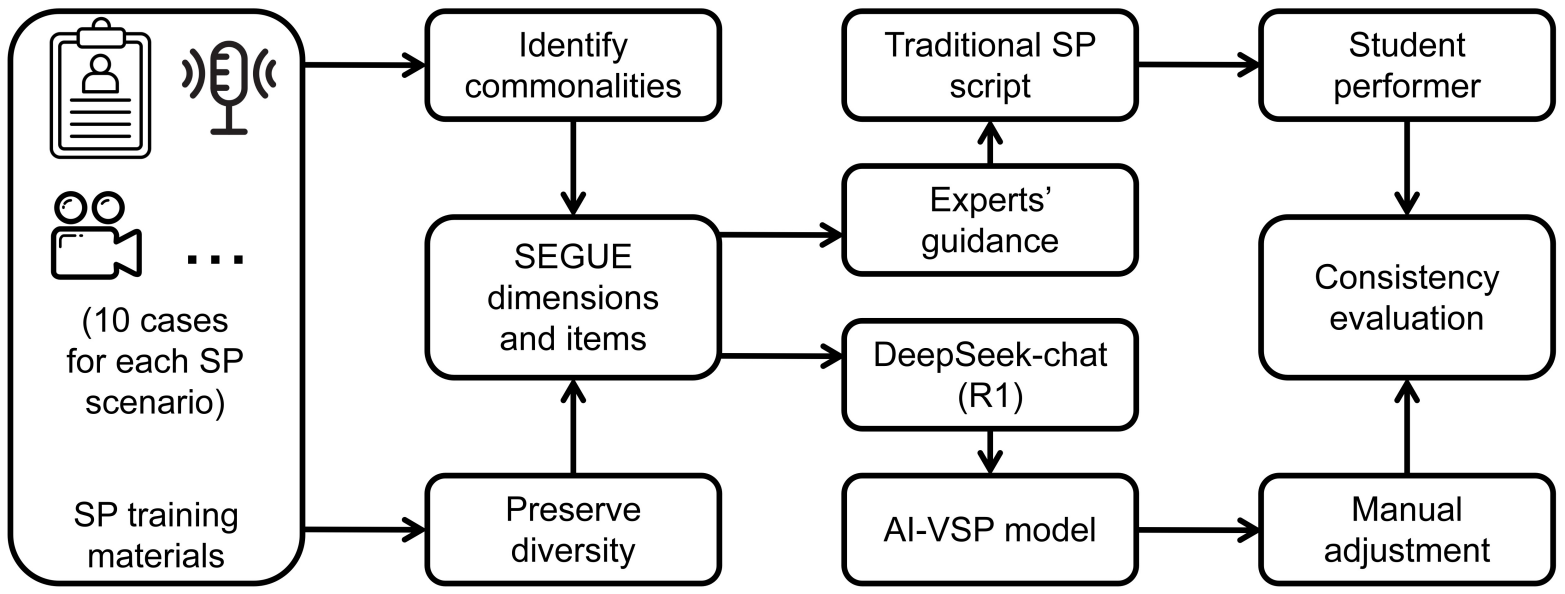
Training flowchart of traditional SP and AI-VSP.

To ensure that AI-VSPs could achieve performance comparable to human SPs, seven senior clinical and teaching experts (Appendix 5) were invited to conduct communication tests with the AI-VSPs based on their experience and the SEGUE assessment criteria. After the first round of testing, the experts provided revision suggestions, which were implemented prior to a second evaluation. Following two rounds of modification, expert voting was conducted. AI-VSPs receiving at least four out of seven “qualified” votes were deemed acceptable for use in the training program. A comparison of the training procedures between AI-VSPs and traditional SPs is illustrated in Figure 1.

### 2.3 Application and system implementation of AI-VSP

An AI-based communication training system was developed in accordance with the SEGUE framework. The system integrated an automatic speech recognition engine (e.g., iFlytek) with a semantic analysis module aligned with the five SEGUE dimensions, enabling the simulation and evaluation of multiple physician–patient communication scenarios.

During training, medical students interacted verbally with the AI-VSP in real time while assuming the role of the physician. Spoken dialogue was automatically captured and transcribed, and key communicative behaviors corresponding to SEGUE scoring items were identified and analyzed based on the transcribed content.

**Figure 2:**
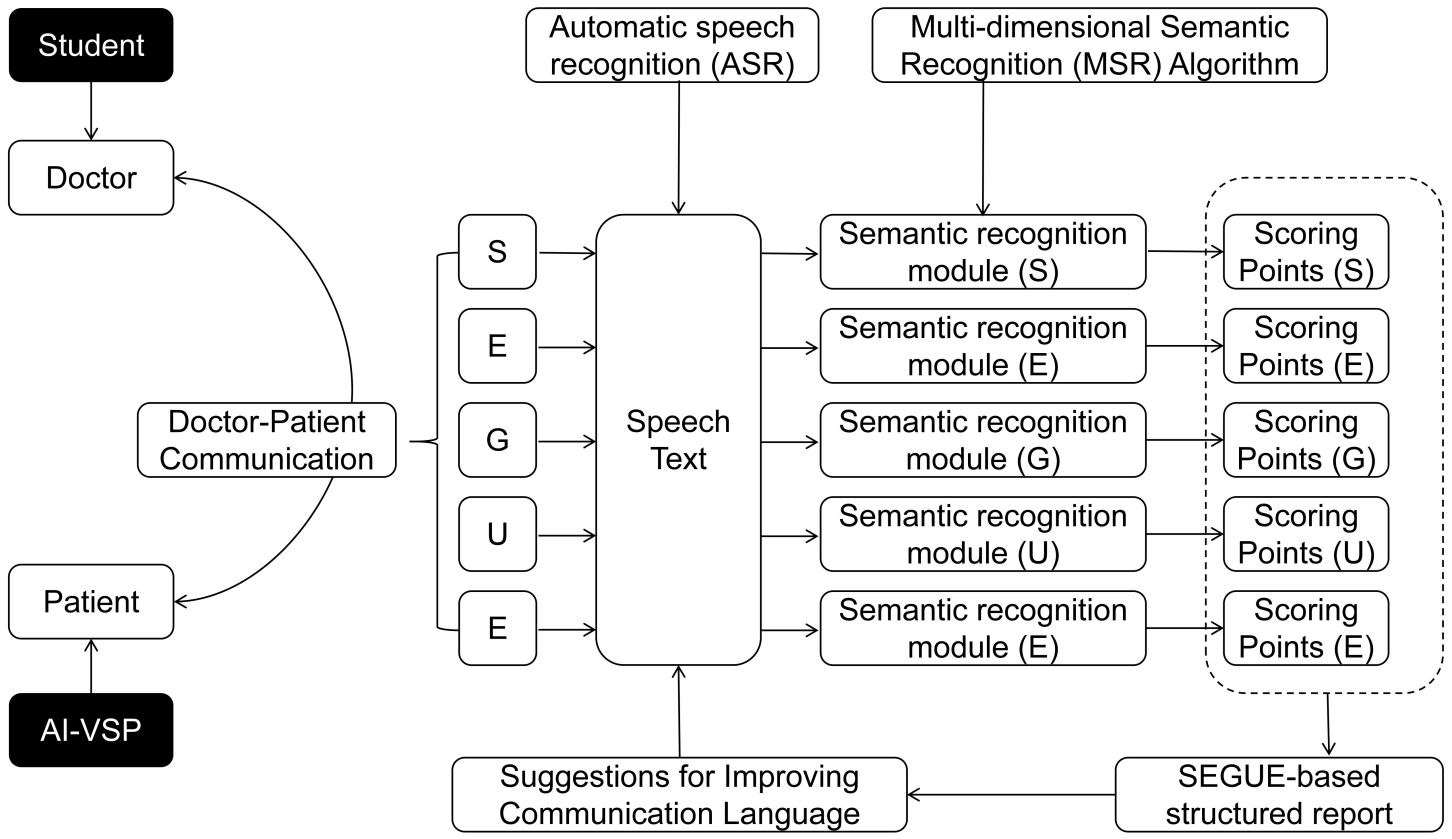
Schematic diagram of AI technology application and system implementation in this study.

After each encounter, the system generated a structured SEGUE-based feedback report, presenting performance scores for each dimension and highlighting representative utterances associated with effective or suboptimal communication. When relevant, example response formulations consistent with SEGUE criteria were provided (Figure 2).

The system was accessed through a web-based interface and supported both classroom-based instruction and independent practice. Core functions included case-based training, simulated assessments, score management, and automated feedback generation, enabling standardized and repeatable communication training.

Interaction with the AI-VSP was conducted exclusively through a real-time conversational interface without visual avatars, facial animation, or synthesized patient imagery, in order to minimize extraneous nonverbal cues. Throughout the interaction, the AI-VSP functioned solely as a patient and did not provide instructional prompts or guidance. All evaluative feedback was delivered only after completion of the encounter.

The AI-VSP system was entirely software-based and did not involve physical mannequins or embodied simulation devices. The system operated via a web-based interface and required stable internet connectivity for cloud-based model access. During the study period, no major internet disruptions were reported, and all training sessions were completed as scheduled without technical interruption.

### 2.4 Sample size calculation and participants

Based on previously published studies conducted in China that applied the SEGUE framework to assess communication skills among medical students, the required sample size was calculated to be 34 participants per group, resulting in a total of 68 participants (two-sided α = 0.05, power = 1 – β = 0.95). Considering a 10% dropout rate, the overall required sample size was estimated to be 76 participants.

In July 2025, a total of 95 senior undergraduate students (4th−5th year) majoring in Clinical Medicine at China Medical University, who were about to begin their clinical internships, were recruited as potential participants.

Inclusion criteria:

- Age ≥ 18 years.
- Able to participate in the 4-week physician–patient communication training program in full.
- Willing to complete the relevant questionnaires.

Exclusion criteria:

- Under 18 years of age.
- Unable to complete the entire training program.
- Unwilling to continue participation.

**Figure 3:**
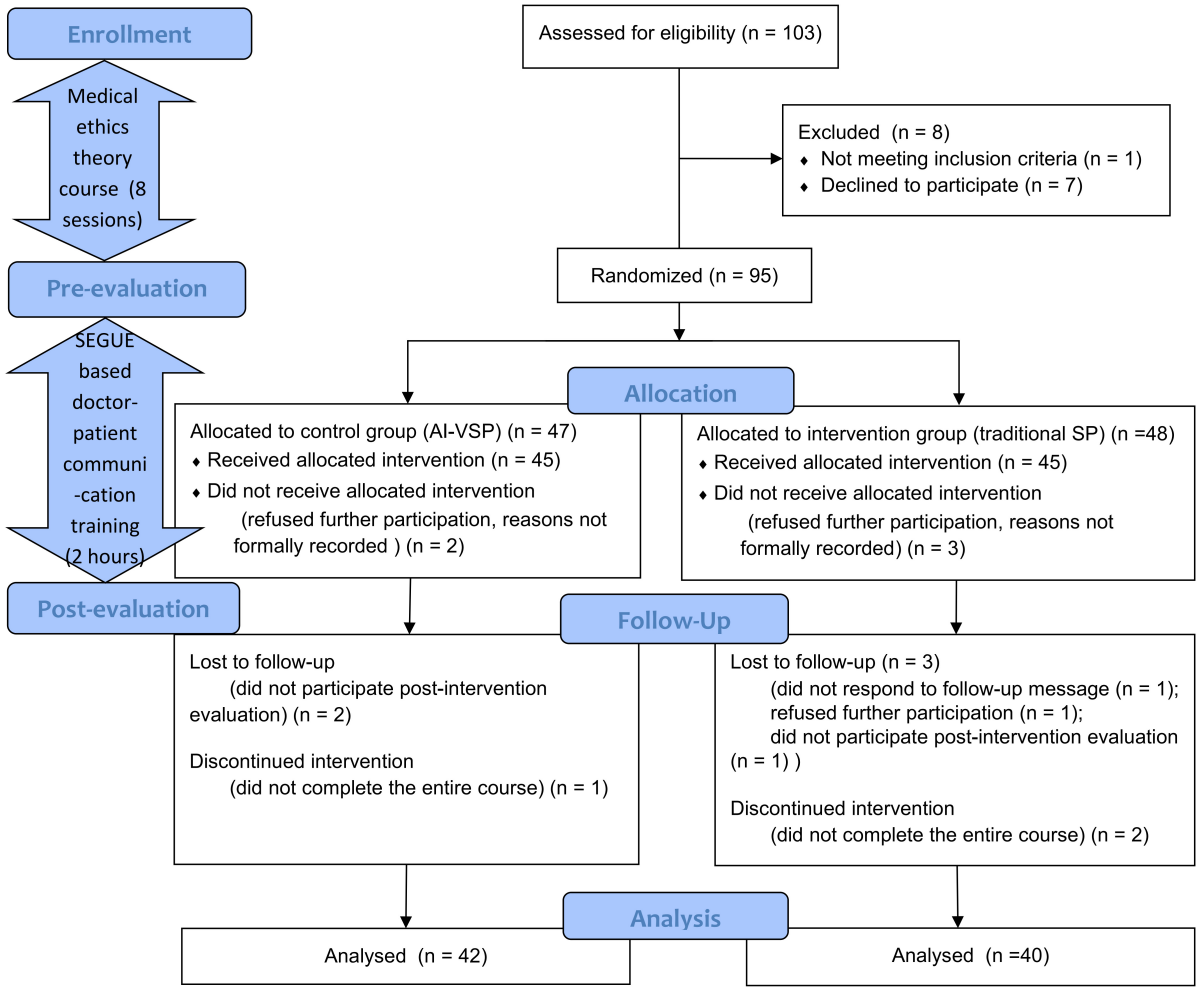
CONSORT diagram of this study.

Ultimately, 82 valid datasets were collected and included in the final analysis (Figure 3).

### 2.5 Study design

This study employed a randomized controlled trial (RCT) design with a parallel mixed-methods approach, integrating quantitative and qualitative data collection, in accordance with the CONSORT 2020 guidelines (20).

All enrolled students first completed a 3–week (8–h) medical ethics theoretical course, followed by a pre-intervention communication skills assessment using human standardized patients (SPs). Based on pre-intervention SEGUE scores, participants were allocated to the intervention or control group through matched-pair randomization to ensure comparable baseline performance.

The intervention group received 2 h of practical communication training using AI-based virtual standardized patients (AI-VSPs), while the control group received 2 h of training using traditional human SPs. In the control group, four SPs were shared among participants (student-to-SP ratio ≈ 11:1), with each SP available for 120 min. Individual training encounters in both groups were limited to 10 min per student.

**Figure 4:**
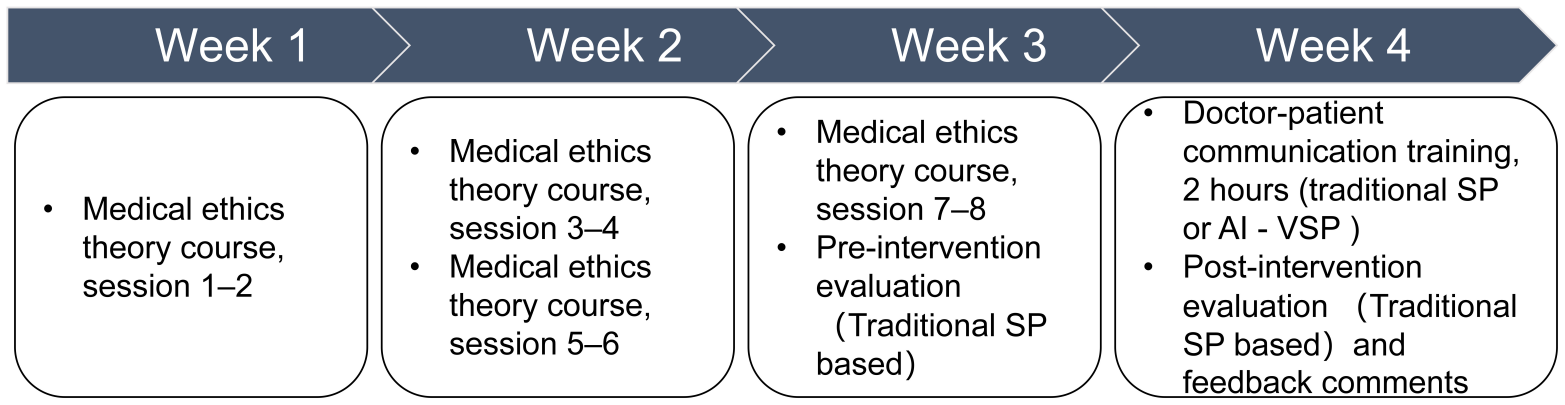
Weekly intervention schedule.

To minimize potential bias related to differences in training duration or practice opportunity, total practical training time was uniformly capped at 2 h for both groups (Figure 4).

All training encounters consisted of bidirectional physician–patient interactions, beginning with natural conversational initiation by either party and ending when the student judged the consultation to be complete or when the 10-min time limit was reached.

In the AI-VSP group, a structured feedback report was generated automatically after each encounter, providing SEGUE-based dimension scores and specific, item-level improvement suggestions. No feedback was provided during the interaction.

In the traditional SP group, no feedback was provided during training, either in real time or after the encounter. Communication performance reports based on independent evaluations by two trained assessors were returned to students on site for both pre-intervention and post-intervention assessments; these reports contained scores only and did not include individualized improvement suggestions.

After completion of the training phase, all participants underwent a post-intervention communication skills assessment using traditional SPs, with students randomly assigned to scenarios. Participants also provided qualitative feedback on their training experience by responding to an open-ended question: “What are your impressions of this training?”

This design was intended to compare two comprehensive communication training models as implemented in routine educational practice.

### 2.6 Scoring criteria

According to the SEGUE scale instructions, content items (items 1–4, 6–11, 16–18, 20–21, 24–25) are scored if they appear at least once during the interaction. In contrast, process items (items 5, 12–15, 19, 22–23) are scored only if the behavior is performed correctly throughout the interaction, meaning that a single omission results in no points for that item.

For example:

- Item 1 (“Politely address the patient”): points are awarded if the student politely addresses the patient at least once during the interaction.
- Item 12 (“Avoid leading or directive questions”): points are deducted if the student asks even one leading or directive question during the interaction.

Scores are first calculated for each SEGUE dimension individually, then summed across all dimensions to obtain a total score. Additionally, total scores for Content and Process are calculated separately.

### 2.7 Statistical analysis

Sample size calculations were performed using G^*^Power version 3.1. All statistical analyses were conducted using SPSS version 25.0.

For quantitative data, continuous variables were analyzed using parametric tests when distributional assumptions were met and non-parametric alternatives otherwise. Normally distributed variables are presented as mean ± standard deviation (SD) and were compared using independent-samples or paired *t*-tests, as appropriate. Non-normally distributed variables were analyzed using the Mann–Whitney U test or Wilcoxon signed-rank test. Categorical variables are presented as frequency (percentage) and compared using the χ^2^ test.

To compare post-intervention communication performance between groups while controlling for baseline differences, analysis of covariance (ANCOVA) was performed with post-intervention SEGUE scores as the dependent variable and pre-intervention scores as covariates, where appropriate.

Regarding training frequency, a single natural communication cycle was defined as beginning with conversational initiation by either party (e.g., “Hello”) and ending with natural closure (e.g., “Thank you, goodbye”). The number of completed training cycles per student was recorded (manually for the traditional SP group and system-extracted for the AI-VSP group). Linear regression analysis was used to examine the association between the number of training cycles and changes in communication skill scores.

Qualitative data from open-ended feedback were analyzed using conventional text analysis procedures. Responses were first coded for meaningful content, then grouped into categories, and the relative proportions of codes and categories were calculated.

All statistical tests were two-sided, with a significance level of α = 0.05. Effect sizes (Cohen's d) were calculated for between-group comparisons to facilitate interpretation of the educational relevance of statistically significant findings.

### 2.8 Ethical considerations

The medical ethics theoretical course involved in this study is a mandatory component of the existing curriculum. However, the SEGUE assessment and standardized patient (SP) training were not part of the official course content and did not contribute to students' grades. Participation was entirely voluntary. After being informed of the study objectives and the intended use of collected data, students provided electronic informed consent and retained the right to withdraw at any time.

Patient case materials used in this study were obtained through secondary use of existing clinical records. All patient information was de-identified, including personal identifiers, voice, and facial features, to protect privacy.

This project was registered as a school-level research project at China Medical University (Project No. YDJG2025040) and received ethical approval from the Ethics Committee of the Fourth Affiliated Hospital, China Medical University (Ethics No. EC-2024-KS-214).

## 3 Results

A total of 82 valid datasets were included in the final analysis, comprising 40 participants in the intervention group (AI-VSP) and 42 participants in the control group (traditional SP). There were no significant between-group differences in gender, age, or academic year at baseline (Table 3). Pre- and post-intervention SEGUE scores were approximately normally distributed, whereas training frequency showed slight deviation from normality but remained approximately symmetric.

**Table 3:** Distribution of socio-demographic characteristics.

Characteristics	Mean (SD)/ *n* (%)		Independent *t*-test/Chi–square	*P*
	**Traditional SP (CG**, ***n*** = **42)**	**AI-VSP (IG**, ***n*** = **40)**		
Age	21.90 (0.82)	21.53 (0.98)	1.90	0.06
Gender				
Male	21 (50.0%)	21 (52.5%)	0.05	0.82
Female	21 (50.0%)	19 (47.5%)		
Grade				
Fourth year	26 (61.9%)	25 (62.5%)	0.003	0.96
Fifth year	16 (38.1%)	15 (37.5%)		

### 3.1 Within-group comparison of communication skills before and after intervention

**Figure 5:**
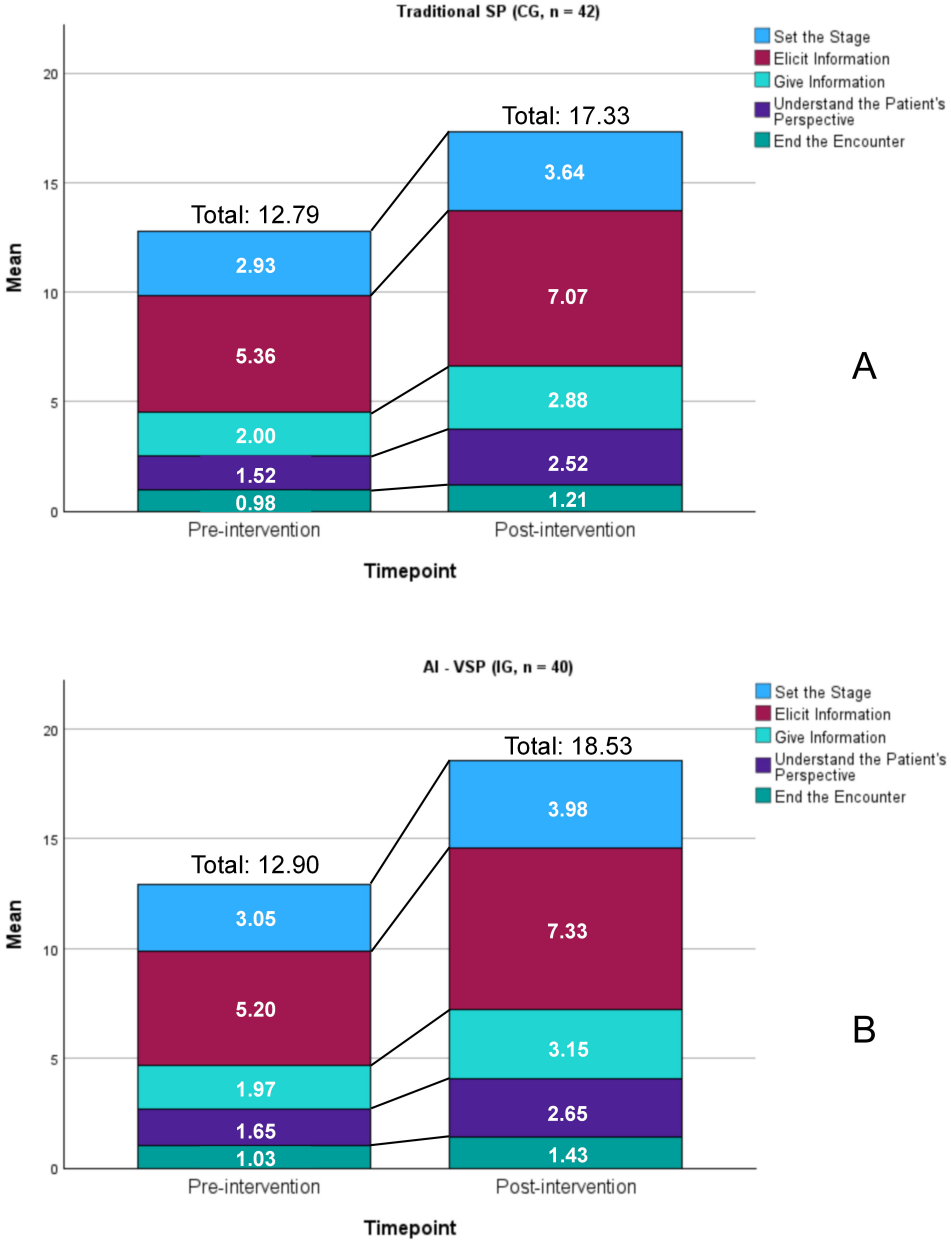
Comparison of pre- and post-intervention mean SEGUE dimension scores. **(A)** traditional SP (CG, *n* = 42); **(B)** AI - VSP (IG, *n* = 40). All, *P* < 0.05.

**Figure 6:**
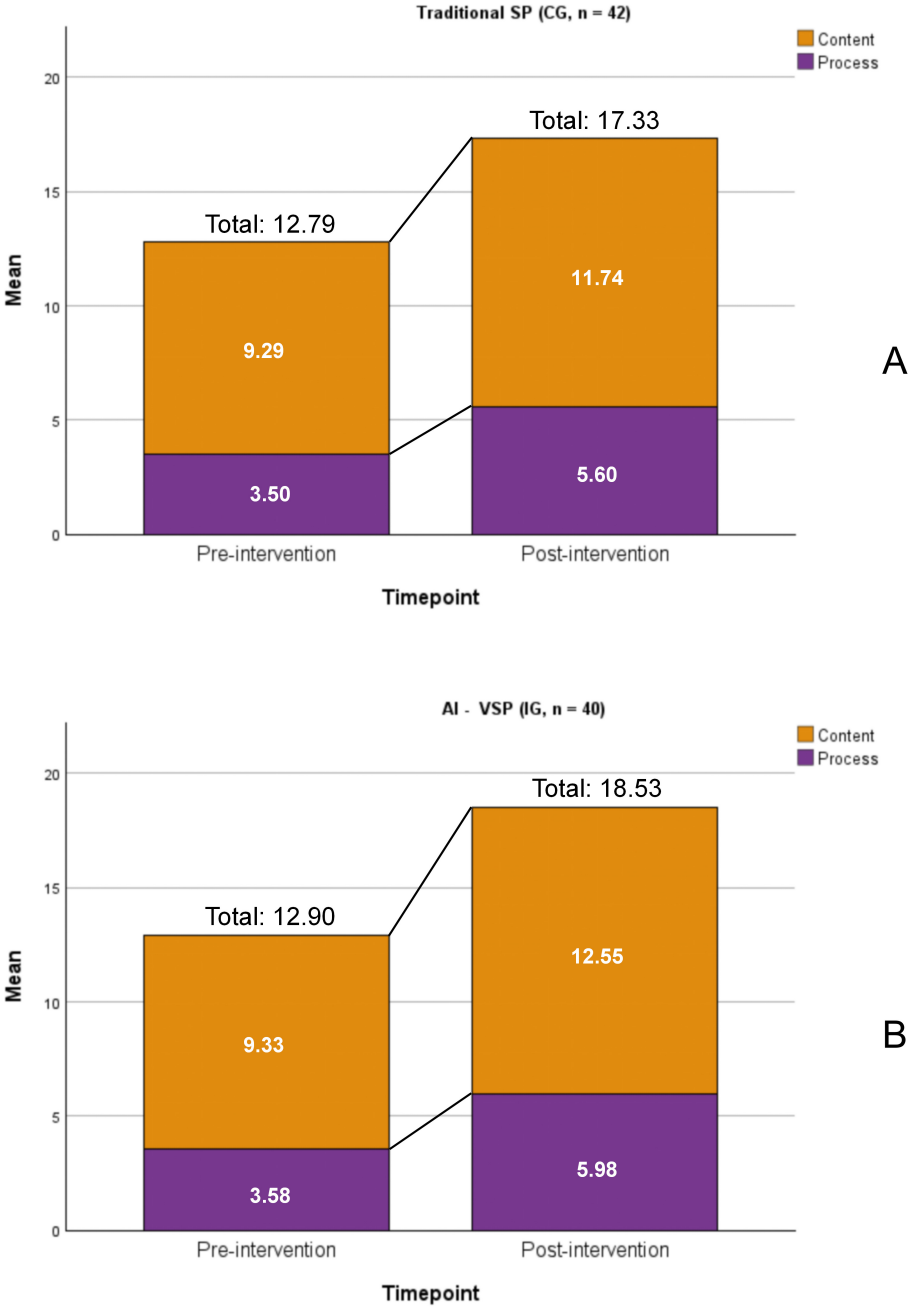
Comparison of pre- and post-intervention mean scores for Content and Process items. **(A)** traditional SP (CG, *n* = 42); **(B)** AI - VSP (IG, *n* = 40). All, *P* < 0.05.

Figures 5, 6 show that, prior to intervention, both the traditional SP (control) group and AI-VSP (intervention) group had mean total SEGUE scores of 12.79 and 12.90 out of 25, respectively. After the intervention, both groups demonstrated significant improvements across all SEGUE dimensions, total scores, and Content and Process sub-scores (all, *P* < .05).

### 3.2 Between-group comparison of communication skills

As shown in Table 4, pre-intervention scores for total SEGUE, individual dimensions, Content, and Process items did not differ significantly between the AI-VSP (intervention) and traditional SP (control) groups.

**Table 4:** Comparison of Pre- and Post-intervention SEGUE scores [Mean (SD)] between groups.

SEGUE dimensions	Question No.	Pre–intervention					Post–intervention					
		**Mean (SD)**		***t1 (df** = **80)***	***p1***	**Cohen's d**	**Mean (SD)**		***t2 (df** = **80)***	***p2***	**Cohen's d**	**ANCOVA** ***P***
		**CG**	**IG**				**CG**	**IG**				
Total	Q1–Q25	12.79 (2.23)	12.90 (2.25)	−0.23	0.82	−0.05	17.33 (2.13)	18.53 (2.51)	−2.32	0.02	−0.51	0.01
Set the Stage	Q1–Q5	2.93 (1.02)	3.05 (1.01)	−0.54	0.59	−0.12	3.64 (1.01)	3.98 (0.80)	−1.65	0.10	−0.36	0.10
Elicit Information	Q6–Q15	5.36 (1.58)	5.20 (1.20)	0.51	0.61	0.11	7.07 (1.46)	7.33 (1.42)	−0.80	0.43	−0.18	0.16
Give Information	Q16–Q19	2.00 (0.88)	1.98 (0.89)	0.13	0.90	0.03	2.88 (0.77)	3.15 (0.83)	−1.52	0.13	−0.34	0.10
Understand the Patient's Perspective	Q20–Q23	1.52 (0.92)	1.65 (0.86)	−0.64	0.52	−0.14	2.52 (0.97)	2.65 (0.74)	−0.66	0.51	−0.15	0.66
End the Encounter	Q24–Q25	0.98 (0.56)	1.03 (0.62)	−0.37	0.71	−0.08	1.21 (0.57)	1.43 (0.68)	−1.54	0.13	−0.34	0.046
Content	Q1–Q4, Q6–Q11, Q16–Q18, Q20–Q21, Q24–Q25	9.29 (1.69)	9.33 (1.76)	−0.10	0.92	−0.02	11.74 (1.38)	12.55 (1.91)	−2.22	0.03	−0.49	0.01
Process	Q5, Q12–Q15, Q19, Q22–Q23	3.50 (1.60)	3.58 (1.38)	−0.23	0.82	−0.05	5.60(1.55)	5.98(1.44)	−1.15	0.25	−0.25	0.30

After the intervention, the AI-VSP group demonstrated statistically higher total scores (18.53 ± 2.51) and Content scores (12.55 ± 1.91) compared with the traditional SP group (17.33 ± 2.13 and 11.74 ± 1.38, respectively; both *P* < .05). The corresponding effect sizes were moderate in magnitude (total score: Cohen's d = −0.51; Content score: Cohen's d = −0.49). When adjusting for baseline scores and training frequency using ANCOVA, the between-group differences in total and Content scores remained statistically significant (both *P* = 0.01). There were no significant differences observed in individual dimension scores or Process scores between the groups.

### 3.3 Relationship between training frequency and SEGUE score improvement

Table 5 shows that the AI-VSP group completed a higher number of training cycles than the traditional SP group (mean ± SD: 4.10 ± 0.67 vs. 3.79 ± 0.52). This difference was statistically significant based on the Mann–Whitney U test (*U* = 639.5, *P* = 0.03), with a small-to-moderate effect size (*r* = 0.25).

**Table 5:** Comparison of mean training frequency between Traditional SP and AI-VSP groups.

Traning frequency	Traditional SP (CG, *n* = 42)		AI-VSP (IG, *n* = 40)		Mann-Whitney U	*P*	*r*
	***n*** **(%)**	**Mean (SD)**	***n*** **(%)**	**Mean (SD)**			
3	11 (26.2%)	3.79 (0.52)	6 (15.0%)	4.10 (0.67)	639.50	0.03	0.25
4	29 (69.0%)		25 (62.5%)				
5	2 (4.8%)		8 (20.0%)				
6	0		1 (2.5%)				

**Figure 7:**
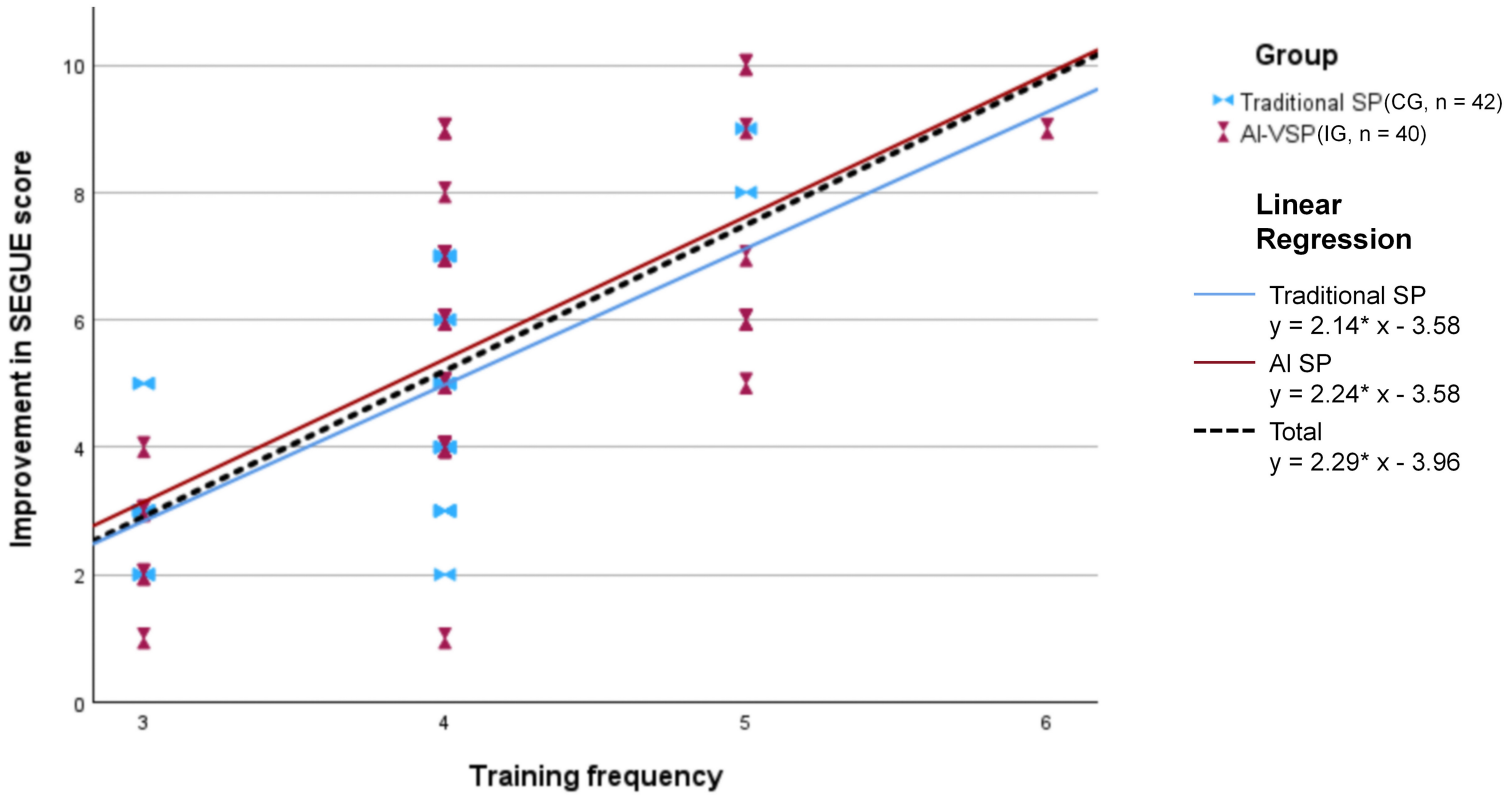
Scatter plot of post-intervention SEGUE score improvement by training frequency, with linear regression lines for each group and the total sample (all, *P* < .001).

Figure 7 presents scatter plots and linear regression trends of training frequency × SEGUE score improvement. Using the overall regression line as reference, the AI-VSP group's regression line was positioned above that of the traditional SP group, indicating a stronger positive association between training frequency and score improvement (all, *P* < .001).

However, when post-intervention SEGUE scores were entered as the dependent variable in an ANCOVA model, with group as the fixed factor and pre-intervention score and training frequency as covariates, the between-group difference was no longer statistically significant (*t* = −1.41, df = 80, *P* =.163).

### 3.4 Text analysis of feedback comments

A total of 78 feedback comments were collected (39 from each group), representing a 95.1% response rate. After excluding non-informative comments such as “Good” or “No comments,” 20 valid comments remained in the AI-VSP group and 24 in the traditional SP group.

Textual coding revealed that the AI-VSP group expressed predominantly positive attitudes, with 91.7% of valid comments containing positive statements, while only 20.8% included negative remarks. In contrast, the traditional SP group showed 75.0% positive and 50.0% negative comments (Table 6).

**Table 6:** Distribution of positive and negative feedback codes in traditional SP and AI-VSP groups.

Traditional SP (CG, *n* = 42)	Frequency	Ratio	AI-VSP (IG, *n* = 40)	Frequency	Ratio
Valid responses	20	47.6%	Valid responses	24	60.0%
Positive codes	15	75.0%	Positive codes	22	91.7%
Sense of realism / interactivity	8	40.0%	Intelligent / realistic	8	33.3%
Deepened understanding / helpful	7	35.0%	Helpful	6	25.0%
Negative codes	10	50.0%	Repeatable / convenient	8	33.3%
Monotony / lack of richness	2	10.0%	Negative codes	5	20.8%
Nervousness	4	20.0%	Training model needs improvement	3	12.5%
Poor effect / difficulty	4	20.0%	Useless / unrealistic	2	8.3%
Invalid responses (No comments/ Good/ Very good, etc)	16	38.1%	Invalid responses (No comments/ Good/ Very good, etc.)	15	37.5%
No responses (Duplicated, incomplete or garbled text, etc.)	6	14.3%	No responses (Duplicated, incomplete or garbled text, etc.)	1	2.5%
Total	42	100.0%	Total	40	100.0%

The AI-VSP group frequently highlighted intelligence, realism, helpfulness, and especially the repeatability and convenience of training, which emerged as the most distinctive theme. Meanwhile, comments from the traditional SP group mainly emphasized realism, interactivity, and deeper understanding, but also reported concerns such as monotony, nervousness during interaction, and limited training effectiveness.

Overall, the AI-VSP group demonstrated higher acceptance and perceived usefulness, particularly due to its ability to support repetitive practice, accessibility, and flexibility, which were less frequently mentioned in the traditional SP group. All comments and coding details are provided in Appendices 6–7.

## 4 Discussion

This study demonstrated that both AI-VSP and traditional SP groups showed overall improvement in communication skills within the SEGUE framework following simulation-based training. To the best of our knowledge, this study is the first to directly compare AI-based virtual standardized patients and traditional standardized patients using SEGUE-based communication skill assessment. The AI-VSP group achieved higher post-intervention overall and content-related performance, whereas no clear differences were observed in other communication dimensions. Within the same training duration, students in the AI-VSP group completed more training cycles, and greater training frequency was associated with larger improvements in communication performance. However, when baseline performance and training frequency were taken into account, the between-group difference was attenuated, suggesting that the observed advantage of AI-VSPs may be largely attributable to their facilitation of more frequent and flexible practice opportunities rather than to the modality itself. Text analysis further indicated that the AI-VSP group received more positive and fewer negative comments than the traditional SP group.

### 4.1 Necessity of simulation-based communication training

Post-intervention SEGUE scores in this study were generally consistent with previous domestic studies (12, 13, 21, 22). However, compared with the average scores reported in the United States, both groups' post-intervention total scores remained lower, reflecting the relative weakness of physician–patient communication education and training in China.

Both within-group and between-group comparisons confirmed the necessity of simulation-based communication training. Pre-intervention, both groups scored only about 50% of the maximum SEGUE score, indicating that traditional 8-h medical ethics courses alone enabled students to complete approximately half of the communication tasks. After just 2 h of practical training, students in both groups completed about 70% of the tasks, showing significant improvement. Within-group analysis showed that all SEGUE dimensions improved significantly, while between-group analysis revealed no significant differences across individual dimensions (all *P* >.05), despite the AI-VSP group achieving a higher total score (*P* = 0.023). These findings indicate that both types of standardized patients can effectively enhance overall physician–patient communication skills. Consistent with the quantitative results, qualitative feedback revealed that 13 students (15.9%) explicitly perceived the training as helpful, as summarized in Table 6. Medicine is inherently practice-based, and physician–patient communication requires hands-on practice, as theoretical instruction alone may not fully achieve the learning objectives (23–25).

### 4.2 Limitations of current physician–patient communication education

Within the SEGUE framework, Content items are fulfilled when required information is expressed at least once, whereas Process items require sustained demonstration throughout the encounter. In this study, post-encounter automated feedback in the AI-VSP system provided clear, item-level guidance that was particularly conducive to improving Content-related behaviors. This design creates an asymmetric alignment with the SEGUE scoring structure, which may preferentially support procedural completeness rather than deeper interactional qualities. Accordingly, observed between-group differences should be interpreted in light of differences in feedback structure and practice support, rather than attributed solely to the training modality itself.

Consistent with this design, the AI-VSP group achieved post-intervention Content scores nearly 1 point higher than the traditional SP group (out of 17 points, *P* = 0.03), whereas no significant between-group difference was observed for Process scores (*P* = 0.254). This pattern suggests more efficient procedural learning, whereby explicit feedback facilitated recall and execution of required informational elements, rather than deeper communicative understanding.

By contrast, Process items emphasize interactional qualities such as empathy, responsiveness, and perspective-taking, which require sustained, context-sensitive behaviors and are less amenable to brief, item-level feedback. Although traditional SPs theoretically offer greater interactional realism, no significant between-group differences were observed for Process scores, and improvements were limited to approximately 2–2.5 additional items compared with pre-intervention performance, consistent with prior reports of constrained short-term gains in higher-order communication skills (26, 27).

Importantly, these findings also reflect a broader limitation of current physician–patient communication and medical ethics education. While simulation-based training can improve procedural completeness and information delivery, core elements of patient-centered communication—such as empathy, perspective-taking, and genuine relational engagement—show limited improvement. This may be attributable to the fact that such competencies are primarily addressed at the level of curricular teaching rather than being explicitly trained, reinforced, or assessed within existing simulation and evaluation frameworks.

### 4.3 Advantages and limitations of AI-VSP

The positive association between SEGUE score improvement and training frequency observed in this study is consistent with prior findings. Existing evidence indicates that AI-enabled simulation can provide convenient, repeatable, and cost-effective training without compromising educational quality compared with traditional approaches (28). Within the same 2-h training duration, students in the AI-VSP group completed, on average, 0.31 more training cycles than those in the traditional SP group (4.10 vs. 3.79 cycles; *P* = 0.03), with a small-to-moderate effect size (*r* = 0.25). This higher training frequency likely contributed to the steeper improvement trajectory observed in the AI-VSP group, underscoring the efficiency and accessibility of AI-VSP–based training. Qualitative findings further supported this interpretation. Text analysis revealed 17% more positive comments and 29% fewer negative comments in the AI-VSP group, with “repeatable/convenient” emerging as the key differentiating theme. With respect to perceived authenticity, realism-related comments were comparable between groups. This may reflect the structured, verbally focused training context rather than true equivalence, as AI-VSPs inherently lack spontaneous nonverbal and affective variability characteristic of human SPs.

However, AI-VSP also has limitations. After adjustment for baseline performance and training frequency, no significant between-group differences in post-intervention SEGUE scores were observed, suggesting that the apparent advantages of AI-VSPs are more likely attributable to greater practice opportunity and structured feedback than to the AI modality itself. In this sense, AI-VSPs primarily function as a facilitator of more efficient or frequent practice rather than as an intrinsically superior training approach. Negative feedback such as “Training model needs improvement” and “Useless/Unrealistic” primarily reflect the current AI-VSP's lack of voice, tone, facial expressions, and body language, which are inherent limitations of current virtual models (26, 29, 30). Additionally, to maintain comparability with traditional SPs, AI-VSP training materials were restricted to a limited set of cases (10 per SP). Despite this constraint, AI-VSPs were able to provide more diverse and case-consistent feedback than traditional SPs, whose portrayals are often generic, as illustrated in Appendix 7.

### 4.4 Costs of using traditional SPs

After the study, follow-up interviews with four traditional SPs and two raters revealed that, beyond objective time costs, mental fatigue was a major concern, and none were willing to repeat the role, as summarized in Table 7. SPs and raters are therefore essentially “single-use” resources, limiting repeatability. Both sides suggested that AI could substitute for human SPs, reflecting a perceived lower cost-effectiveness of traditional SPs.

**Table 7:** Feedback and reflections from performers and raters during the role-playing practice.

Student performer	Personal feelings	Practice duration	Would you be willing to play the role / rate again?
1	I feel they (“doctors”) were quite nervous, probably because their knowledge base isn't sufficient.	2 to 3 days, all performers and raters practiced and discussed together under our guidance	All 4 performers refused. One of them said: “It was also a learning experience for me, and I got to know a lot about this disease. But I don't want to play the role again—I felt anxious seeing how nervous they (‘doctors') were.”
2	Can't this be replaced by the AI? It really feels like a waste of time.		
3	The “doctors” seemed a bit overwhelmed by my emotions, hahaha–		
4	I feel some people's questions have already gone off track.		
Rater	—		—
1	They didn't sound very confident when asking questions—still lacking practical experience. They were stammering and struggling to speak smoothly.		Both raters refused. One of them said: “As a rater, I found it quite meaningful to see the students' growth. But I'd suggest using artificial intelligence for scoring in the future, haha.”
2	The ability to communicate with patients is related to one's general communication skills.		

Logistical coordination was also challenging: scheduling 6 staff members for over 80 students led to missed sessions and participant attrition. Even with time and role adjustments, the SP-to-student ratio reached 1:20, and rater workload doubled, resulting in very brief interactions (under 5 min) in some cases (31), far below the SEGUE standard of 10–15 min. Scaling up to meet the training needs of hundreds of students would require dozens of SPs and raters, further increasing financial and logistical burdens.

## 5 Limitations

This study has several limitations. Participants were senior clinical medical students from a single institution, which may limit generalizability to other learner levels, clinical contexts, or cultural settings. The AI-VSP modules are still under development and cannot fully replicate real patient interactions, potentially affecting study outcomes. Communication skills were assessed solely using the SEGUE framework without corresponding instructional content, which may limit the comprehensiveness of evaluation. Finally, the results reflect short-term skill acquisition and do not address long-term retention or real-world transfer.

## 6 Conclusion

Simulation-based training in physician–patient communication is essential, as current educational approaches exhibit a certain disconnect between theory and practice. Both traditional SPs and AI-VSPs, when applied within the SEGUE framework, can effectively enhance medical students' communication skills. However, the high cost and logistical burden of traditional SPs limit their scalability for widespread use. Although still in need of further refinement, AI-VSPs offer advantages in repeatability and providing feedback on communication completeness, but do not necessarily improve real-world communication competence, such as empathy or relational skills.

## Data Availability

The original contributions presented in this study are included in the article and its Supplementary material. Due to the inclusion of sensitive clinical data and research participant-related information that could potentially identify individuals, the raw data cannot be made publicly available to protect research participant privacy, in line with the ethical approval (Ethics No. EC-2024-KS-214) for this study. Further inquiries regarding the data can be directed to the corresponding authors (Zhong Wang: wz081339@163.com) for legitimate research purposes.
