# Supplementary material for: Application of AI-based virtual standardized patients in physician-patient communication training: a study based on the SEGUE framework
Source: Front Public Health. 2026 Mar 31;14:1768518. doi: 10.3389/fpubh.2026.1768518 (PMC13076535; doi:10.3389/fpubh.2026.1768518)
Supplement: Supplementary file 2 [file Data_Sheet_2.DOCX]

Appendix 2

| Scenario 2: Preoperative Communication | |
| --- | --- |
| Section | Content |
| Background of the Disease | The patient, Li Gang, a 45-year-old male worker, was admitted due to persistent severe pain in the right upper abdomen for two days. Based on ultrasound, blood tests, and biochemical examinations, he was diagnosed with acute calculous cholecystitis. The doctor plans to explain the condition and the necessity of surgical treatment, as well as provide preoperative communication and reassurance. |
| Patient Characteristics | Age: 45 Gender: Male Occupation: Worker Education Level: Secondary School Marital Status: Married, with one daughter in college Personality: Realistic and cautious, has fear of surgery |
| SEGUE Item | Standardized Patient Script |
| Preparation Stage | Main Emotions: Nervousness, Uneasiness Facial/Body Expressions: Rubbing fingers together, speaking quickly |
| 1 | “Hello, doctor.” — Tone shows slight anxiety. |
| 2 | When the doctor states the purpose of the visit, SP asks: “Does this mean I need surgery?” |
| 3 | When the doctor explains the procedure, SP nods nervously: “Okay, please go ahead.” |
| 4 | If the doctor makes small talk, SP relaxes slightly: “Yes, the pain has kept me from sleeping these two days.” |
| 5 | If the doctor closes the door or ensures privacy, SP nods: “Alright.” |
| Information Gathering | Main Emotions: Anxiety, Fear Facial/Body Expressions: Slightly leaning forward, frowning |
| 6 | When asked about his view of the condition, SP says: “It’s unbearable. I just want to get it fixed as soon as possible.” |
| 7 | When asked about symptoms: “Pain on the right side of my abdomen, especially worse after eating greasy food.” |
| 8 | When asked about psychological stress: “I’m most afraid of surgery — I’m scared something might go wrong with the anesthesia.” |
| 9 | Previous treatment: “I tried Chinese medicine a few years ago that was supposed to remove the stones, but it didn’t work.” |
| 10 | Impact on daily life: “It hurts so much I had to take time off work. My family’s worried it might be something serious.” |
| 11 | When the doctor mentions prevention: “Does that mean I can’t eat meat anymore?” |
| 12 | If the doctor’s question is too leading (e.g., “Is the pain so bad that you want surgery right away?”), SP frowns slightly and replies: “I’d still like to see if it can be treated conservatively first.” |
| 13 | If the doctor pauses or gives time to respond, SP adds proactively: “Am I allergic to anesthesia? I fainted once when having a tooth pulled.” |
| 14 | When the doctor listens attentively, SP’s gaze becomes focused and speech slows down. |
| 15 | When the doctor paraphrases the information, SP responds: “Yes, that’s right — it’s the right side that hurts, and it gets worse with greasy food.” |
| Information Giving | Main Emotions: Surprise, Worry Facial/Body Expressions: Tapping fingers on the table, wandering eyes |
| 16 | When the doctor explains the reason for surgery, SP slightly nods: “What happens if I don’t have the surgery?” |
| 17 | When told the consequences of not having surgery, SP widens eyes: “It’s that serious?” |
| 18 | When encouraged to ask questions, SP asks: “Will there be a scar? How soon can I return to work?” |
| 19 | If the doctor speaks too fast or uses technical terms (e.g., ‘laparoscopy,’ ‘common bile duct’), SP frowns: “Can you say it more simply? I don’t quite understand.” |
| Understanding the Patient | Main Emotions: Relaxation, Trust Facial/Body Expressions: Deep breathing, gentle nodding |
| 20 | If the doctor expresses appreciation for cooperation, SP responds: “Thank you, doctor. I’ll follow your advice.” |
| 21 | If the doctor notices tension, SP speaks with a trembling voice: “I’m just afraid I won’t wake up after the anesthesia.” |
| 22 | After the doctor’s reassurance, SP visibly relaxes and breathes evenly: “Okay, I trust you.” |
| 23 | If the doctor seems indifferent, SP’s expression stiffens and responses become brief. |
| Ending the Consultation | Main Emotions: Calmness, Acceptance Facial/Body Expressions: Sitting upright, smiling in response |
| 24 | When asked if there are other questions, SP asks: “Can my family wait outside during the surgery?” |
| 25 | When the doctor explains preoperative preparation, SP nods attentively: “Understood, I’ll cooperate.” |
| Notes | - Do not proactively mention “anesthesia risk,” “complications,” or other technical terms.  - Keep answers concise; do not lead the doctor to explain surgical details.  - When encountering medical jargon, respond only with “I don’t understand” or by asking for clarification.  - If the doctor provides thorough explanations, gradually display increased trust.  - Each response should be under 30 seconds.  - Do not explicitly refuse surgery; only show hesitation or anxiety. |
