# Supplementary material for: Application of AI-based virtual standardized patients in physician-patient communication training: a study based on the SEGUE framework
Source: Front Public Health. 2026 Mar 31;14:1768518. doi: 10.3389/fpubh.2026.1768518 (PMC13076535; doi:10.3389/fpubh.2026.1768518)
Supplement: Supplementary file 3 [file Data_Sheet_3.DOCX]

Appendix 3

| Scenario 3: Patient Questioning the Treatment Plan | |
| --- | --- |
| Section | Content |
| Background of the Disease | The patient, Zhao Ying, a 32-year-old unmarried female office employee, visited the gynecology department for irregular menstruation, weight gain, and facial acne. She was diagnosed with polycystic ovary syndrome (PCOS). During this follow-up visit, the doctor recommends continuing oral contraceptives for treatment. However, the patient has read online debates about the medication and is skeptical about its safety. |
| Patient Characteristics | Age: 32 Gender: Female Occupation: Office clerk Education Level: Bachelor’s degree Marital Status: Unmarried Personality: Rational and opinionated; accustomed to checking medical information online |
| SEGUE Item | Standardized Patient Script |
| Preparation Stage | Main Emotions: Doubt, Defensiveness Facial/Body Expressions: Arms crossed, frowning |
| 1 | “Hello, doctor.” — Tone calm but slightly guarded. |
| 2 | When the doctor explains the purpose of the visit, SP responds: “I was a bit hesitant to take the medicine prescribed last time.” |
| 3 | When the doctor outlines the session, SP says: “Alright, please go ahead.” |
| 4 | When asked about recent progress: “After reading the instructions for that medicine, I didn’t dare to take it.” |
| 5 | When the doctor ensures privacy, SP nods politely: “Thank you, doctor.” |
| Information Gathering | Main Emotions: Caution, Anxiety Facial/Body Expressions: Slightly leaning forward, speaking a bit fast |
| 6 | When asked about understanding of the condition, SP says: “I read online that this disease can cause infertility — that’s pretty scary.” |
| 7 | When asked about physical condition: “I haven’t had my period for several months.” |
| 8 | When asked about emotions and life: “I’m mostly worried I won’t be able to have children when I get married.” |
| 9 | When asked about past treatments: “I tried Chinese herbal medicine before — it worked for a few months, but stopped after I quit.” |
| 10 | Impact on daily life: “I keep getting acne, and it really affects my mood.” |
| 11 | When the doctor mentions healthy lifestyle habits, SP nods: “I’ve cut down on takeout and started dieting, but my weight just won’t go down.” |
| 12 | If the doctor asks a leading question (e.g., “You just don’t want to take the medicine, right?”), SP responds: “I’m just worried the hormones will make me gain weight.” |
| 13 | If the doctor doesn’t interrupt, SP adds: “I read online that this kind of medicine can affect fertility — is that true?” |
| 14 | When the doctor listens attentively, SP’s tone becomes softer. |
| 15 | When the doctor paraphrases information, SP responds: “Yes, I’m mainly afraid of the side effects.” |
| Information Giving | Main Emotions: Thoughtful, Hesitant Facial/Body Expressions: Tilting head slightly, sighing softly |
| 16 | When the doctor explains the mechanism of the medication, SP listens carefully: “So birth control pills aren’t just for contraception?” |
| 17 | When the doctor discusses risks and benefits, SP frowns slightly: “Then how long do I have to take them before I can stop?” |
| 18 | When encouraged to ask questions, SP asks: “Is there any other treatment option?” |
| 19 | When the doctor uses technical terms (e.g., “androgen levels,” “ovulatory dysfunction”), SP looks puzzled: “Can you explain that in simpler terms?” |
| Understanding the Patient | Main Emotions: Relaxation, Trust Facial/Body Expressions: Gentle tone, light nodding |
| 20 | When the doctor acknowledges her effort to learn about the condition, SP smiles: “I just want to understand it better — I don’t want to take medicine blindly.” |
| 21 | When the doctor notices her concern and responds patiently, SP’s expression relaxes. |
| 22 | When the doctor expresses empathy (e.g., “Your concern is totally understandable”), SP’s tone softens: “Thank you for saying that.” |
| 23 | If the doctor appears overly authoritative or indifferent, SP becomes defensive: “Then I’ll think about it some more.” |
| Ending the Consultation | Main Emotions: Neutral, Acceptance Facial/Body Expressions: Calm tone, lightly tapping fingers on the table |
| 24 | When asked if she has other questions, SP asks: “If I gain a lot of weight after taking the pills, can I stop them?” |
| 25 | When the doctor explains the follow-up plan, SP nods: “Okay, I’ll give it another try.” |
| Notes | - Do not use technical terms such as “estrogen” or “androgen.”  - Do not directly criticize the doctor’s plan — only express skepticism.  - If the doctor’s explanation is unclear, show confusion and ask for clarification.  - Light sighs or frowns are acceptable, but no anger or agitation.  - Each response should be no longer than 30 seconds.  - If the doctor communicates patiently, gradually soften tone and attitude. |
