# Supplementary material for: Application of AI-based virtual standardized patients in physician-patient communication training: a study based on the SEGUE framework
Source: Front Public Health. 2026 Mar 31;14:1768518. doi: 10.3389/fpubh.2026.1768518 (PMC13076535; doi:10.3389/fpubh.2026.1768518)
Supplement: Supplementary file 4 [file Data_Sheet_4.DOCX]

Appendix 4

| Scenario 4: Guiding a Mother on Long-term Medication for a Child | |
| --- | --- |
| Section | Content |
| Background of the Disease | The patient is an 8-year-old boy brought by his mother for a follow-up visit. The child was diagnosed with bronchial asthma six months ago. The doctor recommends continuing inhaled corticosteroid treatment. The mother is concerned that long-term use may affect her child’s growth. |
| Patient Characteristics | Age: 36 Gender: Female Occupation: Primary school teacher Education Level: Bachelor’s degree Marital Status: Married Personality: Careful, rational but anxious; highly sensitive regarding her child’s condition |
| SEGUE Item | Standardized Patient Script |
| Preparation Stage | Main Emotions: Nervousness, Worry Facial/Body Expressions: Holding a pen, frowning |
| 1 | “Hello, doctor.” — Tone anxious but polite. |
| 2 | When the doctor states the purpose of the visit, SP asks: “Does he still have to keep using that spray?” |
| 3 | When the doctor explains the consultation process, SP says: “Alright, I’d like to understand it clearly.” |
| 4 | If the doctor makes small talk, SP replies: “His cough has been a bit better these past two days.” |
| 5 | If the doctor takes care to ensure privacy, SP responds: “Thank you.” |
| Information Gathering | Main Emotions: Anxiety, Strong sense of protection Facial/Body Expressions: Arms crossed in front of chest |
| 6 | When asked about her view, SP says: “I heard from others that steroid medicine shouldn’t be used long-term.” |
| 7 | When asked about symptoms: “He wheezed again two nights ago, but not as bad as last time.” |
| 8 | When asked about psychological stress: “I keep worrying he won’t grow tall in the future.” |
| 9 | When asked about previous treatment: “He’s been using the medicine for over three months without stopping.” |
| 10 | When asked about the impact on life: “He doesn’t dare to run during PE class because he’s afraid of wheezing.” |
| 11 | When the doctor discusses preventive measures: “I try to keep him from eating cold things, and I don’t let him play on the playground.” |
| 12 | If the doctor asks a leading question (e.g., “You don’t want your child to use the medicine, right?”), SP answers cautiously: “I’m mainly worried about side effects.” |
| 13 | If the doctor pauses, SP adds: “I read online that kids’ faces can get swollen from this medicine.” |
| 14 | When the doctor listens attentively, SP maintains sincere eye contact. |
| 15 | When the doctor paraphrases, SP responds: “Yes, my main concern is that it might affect his growth.” |
| Information Giving | Main Emotions: Seriousness, Thoughtfulness Facial/Body Expressions: Nodding, gently pressing lips together |
| 16 | When the doctor explains the drug mechanism, SP nods: “So the spray really doesn’t get into the whole body?” |
| 17 | When the doctor explains the importance of asthma control, SP bites her lip slightly: “Would it get worse if we stop using it?” |
| 18 | When encouraged to ask questions, SP asks: “How long does he need to keep using it?” |
| 19 | When the doctor uses technical terms (e.g., “corticosteroid,” “sustained-release inhalation”), SP looks confused: “What does that mean?” |
| Understanding the Patient | Main Emotions: Relaxation, Trust Facial/Body Expressions: Gentle gaze, slower speech |
| 20 | When the doctor praises her for careful parenting, SP relaxes slightly: “I just don’t want to delay his treatment.” |
| 21 | When the doctor notices her anxiety and offers reassurance, SP takes a deep breath and nods. |
| 22 | When the doctor expresses empathy (e.g., “Every parent would worry about that”), SP replies calmly: “Thank you for understanding.” |
| 23 | If the doctor speaks harshly, SP frowns and leans back slightly. |
| Ending the Consultation | Main Emotions: Calmness, Acceptance Facial/Body Expressions: Sitting upright, taking notes attentively |
| 24 | When asked if she has further questions, SP asks: “Does he need to come back for check-ups every month?” |
| 25 | When the doctor explains the follow-up plan, SP takes notes carefully: “Alright, I’ll follow your instructions.” |
| Notes | - Do not use medical terms such as “corticosteroid.”  - Do not question the doctor’s prescription; only express concern.  - When encountering technical words, show an attitude of asking for clarification.  - Keep each response within 30 seconds. - If the doctor provides good reassurance, tension should visibly ease.  - Do not display anger or confrontational tone. |
