# Supplementary material for: Application of AI-based virtual standardized patients in physician-patient communication training: a study based on the SEGUE framework
Source: Front Public Health. 2026 Mar 31;14:1768518. doi: 10.3389/fpubh.2026.1768518 (PMC13076535; doi:10.3389/fpubh.2026.1768518)
Supplement: Supplementary file 5 [file Data_Sheet_5.DOCX]

| Appendix 5. Expert Characteristics Involved in AI VSP Evaluation | | | | | | | |
| --- | --- | --- | --- | --- | --- | --- | --- |
| Expert ID | Gender | Age | Title & Supervisor Level | Specialty | Years of Clinical Experience | Years of Teaching Experience | Years of Medical Ethics Teaching/Guidance |
| 1 | Female | 53 | Chief Physician & Master’s Supervisor | Internal Medicine | 25 | 23 | 20 |
| 2 | Male | 49 | Associate Chief Physician &  Master’s Supervisor | Surgery | 20 | 20 | 10 |
| 3 | Female | 53 | Chief Physician & Master’s Supervisor | General Medicine | 22 | 20 | 12 |
| 4 | Male | 58 | Chief Physician & PhD Supervisor | Neurology | 24 | 21 | 21 |
| 5 | Female | 49 | Associate Chief Physician &  Master’s Supervisor | Pediatrics | 18 | 15 | 10 |
| 6 | Male | 55 | Chief Physician & Master’s Supervisor | Cardiovascular Medicine | 26 | 26 | 23 |
| 7 | Female | 50 | Chief Physician & Master’s Supervisor | Nursing & Medical Humanities | 21 | 21 | 15 |
