# Supplementary material for: Application of AI-based virtual standardized patients in physician-patient communication training: a study based on the SEGUE framework
Source: Front Public Health. 2026 Mar 31;14:1768518. doi: 10.3389/fpubh.2026.1768518 (PMC13076535; doi:10.3389/fpubh.2026.1768518)
Supplement: Supplementary file 6 [file Data_Sheet_6.DOCX]

| Appendix 6 | |  | | |  | |
| --- | --- | --- | --- | --- | --- | --- |
| Traditional SP (CG) participant responses | | Positive codes | | Negative codes | | |
|  |  | Sense of realism / interactivity | Deepened understand-  ing / helpful | Monotony / lack of richness | Nervous-  ness | Poor effect / difficulty |
| 1 | I didn’t feel it was very effective, as the instructor might not be able to address everyone’s questions. |  |  |  |  | √ |
| 2 | During the regular standardized patient (SP) communication training, I could strongly feel the realism of human interaction — the patient’s expressions and tone were very close to real clinical situations, allowing me to naturally practice communication flow and empathy. However, the scenarios were somewhat limited, and each simulation was relatively costly. It would be more rewarding if more case types could be included. | √ |  | √ |  |  |
| 3 | More friendly and realistic. | √ |  |  |  |  |
| 4 | The “patients” acted very well, but were a bit too enthusiastic — they kept urging us to hurry because they were worried we might not finish on time. | √ |  |  |  |  |
| 5 | The format was engaging and effectively enhanced communication skills. The strong interactivity made it more helpful than purely theoretical learning. | √ | √ |  |  |  |
| 6 | Participating in the regular SP communication training allowed me to practice communication skills in simulated real doctor–patient interactions — such as explaining conditions clearly and comforting patients. This training was very close to real clinical practice, helping me improve communication with different types of patients and deepening my understanding of how effective communication benefits diagnosis and treatment. It provided valuable experience for my future clinical work. | √ | √ |  |  |  |
| 7 | Deeply inspired. |  | √ |  |  |  |
| 8 | Sometimes I felt a bit nervous. |  |  |  | √ |  |
| 9 | Still unable to completely simulate real patients. |  |  | √ |  |  |
| 10 | Using standardized patients helped me strengthen basic clinical skills and I greatly benefited from it. |  | √ |  |  |  |
| 11 | Very nervous. |  |  |  | √ |  |
| 12 | Strong sense of experience. | √ |  |  |  |  |
| 13 | The effect was good, but I’d like to try training with virtual standardized patients. |  | √ |  |  |  |
| 14 | Very realistic — it feels immersive. | √ |  |  |  |  |
| 15 | Highly educational and helped me remember the related knowledge more effectively. |  | √ |  |  |  |
| 16 | Difficult. |  |  |  |  | √ |
| 17 | Needs improvement. |  |  |  |  | √ |
| 18 | Participating in standardized patient communication training enabled me to make a cognitive leap from being a “technical operator” to becoming a “warm and empathetic doctor” .I gained far more than expected. |  | √ |  |  |  |
| 19 | Tight schedule. |  |  |  | √ |  |
| 20 | Although regular SPs are closer to clinical reality and provide hands-on experience, they can be challenging for undergraduate medical students — communication with SPs can be difficult. Moreover, due to hardware and software limitations, undergraduates have limited access to high-quality virtual SPs. | √ |  |  |  | √ |
