# Supplementary material for: Application of AI-based virtual standardized patients in physician-patient communication training: a study based on the SEGUE framework
Source: Front Public Health. 2026 Mar 31;14:1768518. doi: 10.3389/fpubh.2026.1768518 (PMC13076535; doi:10.3389/fpubh.2026.1768518)
Supplement: Supplementary file 7 [file Data_Sheet_7.DOCX]

| Appendix 7 | |  | | |  | |
| --- | --- | --- | --- | --- | --- | --- |
| AI-VSP (IG) participant responses | | Positive codes | | | Negative codes | |
|  |  | Intelligent / realistic | Helpful | Repeatable / convenient | Training model needs improvement | Useless / unrealistic |
| 1 | It was really helpful, I liked it! |  | √ |  |  |  |
| 2 | The virtual standardized doctor–patient communication experience was practical and profound. “Standardization” is a framework of empathetic norms rather than a mechanical process. It accurately reproduced key challenges such as information gaps and emotional reassurance. The virtual environment allows repeated trial and reflection, helping learners quickly master communication techniques such as “listen first, then respond,” and better understand that doctor–patient communication requires warmth and patience through mutual collaboration. | √ |  | √ |  |  |
| 3 | Very intelligent — it reproduced medical histories more comprehensively than regular SPs. | √ |  |  |  |  |
| 4 | Compared with SPs, I felt less nervous when talking to the virtual SP. | √ |  |  |  |  |
| 5 | Very intelligent. | √ |  |  |  |  |
| 6 | The training helped improve my doctor–patient communication skills. |  | √ |  |  |  |
| 7 | More training sessions should be added. |  |  |  | √ |  |
| 8 | The virtual patient training model is more convenient and allows practice anytime. |  |  | √ |  |  |
| 9 | Hope to build more diverse training models. |  |  |  | √ |  |
| 10 | Technology empowers compassionate medicine. Through the practice of virtual standardized patients, I deeply felt the innovation technology brings to medical education. It breaks the limits of time and space, allowing medical students to repeatedly practice interviewing and decision-making in a zero-risk environment, greatly enhancing learning flexibility and tolerance for error. This highly realistic interaction not only strengthened our clinical reasoning and communication skills but also allowed us to experience the responsibility and commitment required of a doctor through virtual “life-and-death” scenarios. Technology here is not a cold tool but a bridge carrying humanistic care — preparing us to face every real life with more confidence and readiness. | √ |  | √ |  |  |
| 11 | A large number of dialogue options are stored — stronger than live role-playing. | √ |  |  |  |  |
| 12 | More flexible. |  |  | √ |  |  |
| 13 | Very helpful for my learning. |  | √ |  |  |  |
| 14 | Extremely helpful — helped me understand the difference between real clinical practice and what’s on paper. |  | √ |  |  |  |
| 15 | Helpful, very good. |  | √ |  |  |  |
| 16 | Realistic. | √ |  |  |  |  |
| 17 | Fast and convenient training, realistic scenarios, and improved my communication skills. | √ |  | √ |  |  |
| 18 | Very accessible. |  |  | √ |  |  |
| 19 | The doctor–patient communication training helped me better understand real clinical interactions and master proper communication methods. |  | √ |  |  |  |
| 20 | The virtual simulated patient provides more operational flexibility. |  |  | √ |  |  |
| 21 | Totally useless. |  |  |  |  | √ |
| 22 | Not as vivid or three-dimensional as real human interaction. |  |  |  |  | √ |
| 23 | More cost-effective than using real standardized patients. |  |  | √ |  |  |
| 24 | Can practice anytime, anywhere, without restrictions. |  |  | √ |  |  |
